# MicroRNA-532-3p Suppresses Malignant Behaviors of Tongue Squamous Cell Carcinoma *via* Regulating CCR7

**DOI:** 10.3389/fphar.2019.00940

**Published:** 2019-08-29

**Authors:** Cuijuan Feng, Hyon Il So, Shoucheng Yin, Xingzhou Su, Qiang Xu, Simin Wang, Weiyi Duan, Enjiao Zhang, Changfu Sun, Zhongfei Xu

**Affiliations:** ^1^Department of Orthodontics, School of Stomatology, China Medical University, Shenyang, China; ^2^Department of Oral and Maxillofacial Surgery, Pyongyang Medical College, Kim IL Sung University, Pyongyang, North Korea; ^3^Department of Oral and Maxillofacial Surgery, School of Stomatology, China Medical University, Shenyang, China

**Keywords:** microRNA-532-3p, CCR7, tongue squamous cell carcinoma, proliferation, migration, invasion, apoptosis

## Abstract

To provide better therapeutic avenues for treating tongue squamous cell carcinoma (TSCC), a series of experiments about the effects of microRNA (miR)-532-3p on TSCC malignant behaviors were carried out. The result showed that miR-532-3p was down-regulated and C-C chemokine receptor 7 (CCR7) was up-regulated in the tumor tissues compared with those in the paired paratumor tissues. Further, expression of miR-532-3p was detected in four TSCC cell lines, TSCCA, TCA8113, CAL-27, and SCC-25. The miR-532-3p mimics and inhibitor were transfected into the CAL-27 and TCA8113 cell lines which were the relatively lowest and highest miR-532-3p expressions, respectively. It was found that the overexpression of miR-532-3p suppressed TSCC cell proliferation, migration, invasion, and promoted apoptosis *in vitro*, whilst the knockdown of miR-532-3p reversed these behaviors. The bioinformatics predicted that CCR7 was a downstream gene of miR-532-3p, which was confirmed *via* luciferase assay. Following, the decline of CCR7 in the miR-532-3p mimics group and the rise of CCR7 in the miR-532-3p inhibitor group were also verified. In addition, enhanced cell proliferation, migration and invasion induced by CCR7 were partly restrained by miR-532-3p in TSCC cell. Meanwhile, miR-532-3p attenuated tumourigenesis *in vivo* due to the reduction of tumor volume and Ki-67 positive rate and the increase of apoptotic cells. Taken together, these findings reveal a pivotal role for the miR-532-3p/CCR7 axis in regulating TSCC, and this novel axis could be suitable for therapeutic intervention in TSCC disease.

## Introduction

Tongue cancer (TC) is not only one of the most common carcinomas of head and neck, but also an oral cavity malignancy threatening human health (Yang et al., 2008; Zhang et al., 2019). According to the statistics, its death rate accounts for about one-third to two-thirds of oral cancer. Among TC, tongue squamous cell carcinoma (TSCC) occupies the majority, and the proportion of tongue adenocarcinoma is less. Despite great progress being made in the past few decades regarding the treatment of TC, the survival rate of TC patients is still unsatisfactory, which is a real challenge (Kokemueller et al., 2011). At present, surgical treatment, radiation therapy, systemic chemotherapy and targeted therapy are still the main treatments for TC. However, surgical treatment brings a lot of inconvenience to people and degrades the quality of life because the tongue is an important vocal organ. In addition, TC usually accompanies the invasion of lymph and distant metastasis (Gorsky et al., 2004; Wang et al., 2011). Therefore, we urgently need to carry out deeper research on the molecular mechanism of TC and make breakthrough progress on the basis of conservative treatment.

MicroRNAs (miRNAs), small non-coding RNAs, play considerable roles in the development of cancer. For instance, miR-196a-5p is a potential prognostic marker of delayed lymph node metastasis in early stage TSCC (Maruyama et al., 2018). It is found that miR-488 is significantly decreased in TSCC tissues and cell lines. Further, knockdown of miR-488 promotes invasion (Shi et al., 2018). Hsa-miR-485-5p reverses epithelial to mesenchymal transition (EMT) and promotes cisplatin-induced cell death by targeting PAK1 in TSCC (Lin et al., 2017). MiR-9 induces cell arrest and apoptosis of oral squamous cell carcinoma *via* CDK4/6 pathway (Shang et al., 2018).

MiR-532-3p is a highly conserved miRNA in many species. There is growing evidence indicating that miR-532-3p serves as a tumor promoter or suppressor in multiple human cancers, such as gastric cancer, liver cancer, renal cancer and so on (Xu et al., 2016; Zhai et al., 2018; Han et al., 2019). However, the biological role of miR-532-3p in TC has not been clarified. Meanwhile, the bioinformatics prediction suggests that C-C chemokine receptor 7 (CCR7) may be a potential target gene for miR-532-3p, which is likely to be regulated by miR-532-3p. CCR7 binds to the C-C chemokine ligand 19 or 21, following activates B cells and T cells to regulate immune responses. CCR7 over-expression is associated with larger primary tumors, deeper lymphatic invasion and poorer survival rates in breast cancer (Tutunea-Fatan et al., 2015). Research reported that CCR7 facilitates invasion and migration of TSCC, as well as lymphatic metastasis *in vivo* (Xia et al., 2015).

Thus, the objective of this study is to illuminate the effect of miR-532-3p in TSCC, whilst the CCR7 acts as a potential target gene for miR-532-3p. Our study adds new elements to the multifaceted role of miR-532-3p in TSCC malignancy by revealing a novel role of miR-532-3p/CCR7 axis in TSCC-associated malignant behavior that might be relevant to future therapies.

## Materials and Methods

### Human Specimens

All surgical specimens (paired tumor and paratumor tissues) were collected from patients with TC in the School of Stomatology, China Medical University. All fresh samples were immediately preserved in liquid nitrogen to protect the protein or RNA from degradation. This study was approved by School of Stomatology, China Medical University ethics committee and written informed consent was obtained from all patients.

### Cell Culture and Cell Transfection

The four TSCC cell lines TSCCA, TCA8113, CAL-27, and SCC-25 were obtained from Procell Life Science & Technology (Wuhan, China). TSCCA and TCA8113 cells were cultured in RMPI 1640 (Gibco, USA) plus 10% fetal bovine serum (FBS, Hyclone, USA). CAL-27 and SCC-25 were cultured in Dulbecco’s modified Eagle’s medium (DMEM, Gibco, USA) plus 10% FBS and DMEM/F12 (Gibco, USA) plus 10% FBS, respectively. All cell lines were incubated in a cell incubator at 37°C in 5% CO_2_.

MiR-532-3p mimics/NC mimics and miR-532-3p inhibitor/NC inhibitor were transfected in CAL-27 and TCA8113 using Lipofectamine 2000 (Invitrogen, USA), respectively. MiR-532-3p mimics and CCR7/vector were co-transfected in CAL-27 using Lipofectamine 2000. The co-transfected cells were cultured in complete medium (DMEM+10% FBS+350 ng/ml CCL21). In addition, the CAL-27 cells were divided into two groups, Control and CCL21 (medium contains 350 ng/ml CCL21).

### RNA Isolation and Real-Time PCR

The total RNA was extracted from tissues or cultured cells using RNAsimple reagent (Tiangen, Beijing, China) according to the manufacturer’s instructions. The concentration and purity of RNA was determined using a NANO 2000 Spectrophotometer (Thermo Fisher Scientific, USA). A specific loop primer, 5’- GTTGGCTCTGGTGCAGGGTCCGAGGTATTCGCACCAGAGCCAACTGCAAGT-3’ was used for extending miR-532-3p. Reverse transcription of RNAs was performed with M-MLV reverse transcriptase (Tiangen) and the cDNA was synthesized. Real-time PCR was performed with SYBR Green (Solarbio, Beijing, China). The expression level of miR-532-3p was normalized to endogenous small nuclear RNA U6. Data were analyzed using the 2^–ΔΔCt^ method. The primer sequences were listed as follows: hsa-miR-532-3p mimics, forward, 5’- CCUCCCACACCCAAGGCUUGCA-3’; reverse, 5’- CAAGCCUUGGGUGUGGGAGGUU-3’.

### Western Blot Analysis

Tissues and cells were lysed in RIPA lysis buffer (Solarbio). Lysates were centrifuged at 10,000*g* at 4°C for 5 min. The supernatant was collected. The protein concentration was detected using BCA kit (Solarbio). The proteins were separated by sodium dodecyl sulfate-polyacrylamide gel electrophoresis (SDS-PAGE, Solarbio) and transferred to polyvinylidene difluoride (PVDF) membrane (Millipore, USA). The membranes were blocked with skimmed milk, incubated with primary antibodies at 4°C overnight, followed by incubation with horseradish peroxidase-conjugated secondary antibodies at 37°C for 1 h. Thereupon, the ECL (Solarbio) were added into membranes. The antibody information was listed in **Table 1**.

**Table 1:** Antibody information.

Primary antibodies	Dilution rate	Secondary antibodies	Dilution rate
Ki-67, ABclonal, China	1: 1000	goat anti-rabbit IgG-HRP, Solarbio, China	1: 3000
caspase-3, CST, USA	1: 1000		1: 3000
PARP, CST	1: 1000		1: 3000
cyclin D1, ABclonal	1: 500		1: 3000
cyclin B1, proteintech, China	1: 2000		1: 3000
PTEN, proteintech	1: 500		1: 3000
p-AKT, CST	1: 1000		1: 5000
AKT, CST	1: 1000		1: 5000
E-cadherin, proteintech	1: 2000		1: 3000
vimentin, proteintech	1: 2000		1: 3000
MMP-2, proteintech	1: 500		1: 3000
MMP-9, proteintech	1: 500		1: 3000
CCR7, proteintech	1: 500		1: 3000
GAPDH, proteintech	1: 10000	goat anti-mouse IgG-HRP, Solarbio	1: 3000

### CCK-8 Assay

The cells (2.5×10^4^ cells/ml) were seeded into 96-well plates (200 μl/well) and incubated in a incubator containing 5% CO_2_ at 37°C for 0, 24, 48, and 72 h. Ten μl CCK-8 per well was added into cells for 2 h, and the OD values were detected at 450 nm. Particularly, CCK-8 assay of the cells in Control and CCL21 groups was performed only at 48 h.

### Cell Cycle and Apoptosis Analysis

At 48 h post-transfection, cells were harvested and washed with phosphate-buffered saline (PBS) for two times. For cell cycle analysis, the cells were fixed with 70% ethanol at 4°C for 2 h. Fixed cells were washed with PBS, treated with dye buffer, mixed with propidium iodide, and added into RNase A for 30 min in the dark. The cells were analyzed with fluorescence-activated cell sorting (FACS) by flow cytometry (Aceabio, USA). For apoptosis analysis, an Annexin V-FITC Apoptosis Detection Kit (Beyotime, China) was used according to the manufacturer’s instructions 48 h after transfection. Apoptosis was analyzed with FACS.

### Wound Healing and Transwell Assays

Prior to the wound healing assay, the medium was changed to serum-free medium and treated with 1 μg/ml of mitomycin C for 1 h. Wounds were made with a 200 μl pipette tip. After washing the cell surface with serum-free medium, the cell was observed under microscope and taken images according to the group. After culturing for 24 h, the migration distance was statistic.

The cell invasion assays were carried out using matrigel-coated membranes in 24-well transwell chamber (Corning, USA). The cells (5×10^4^ cells/well) were cultured into the upper chamber with serum-free medium, and the lower chamber contained complete medium. Following 24 h incubation, the cells were fixed with 4% paraformaldehyde and stained with 0.4% crystal violet. The number of cells that invaded to the underlying membrane was counted under a microscope (OLYMPUS, Japan).

### Luciferase Assay

The 381/400 bp wild type (WT) 1/2 3’-UTR of CCR7 containing miR-532-3p binding site was amplified by PCR and inserted into the NheI/SalI sites to generate CCR7 WT1/2. The complementary sequence for miR-532-3p seed sequence in CCR7 3’-UTR was mutated using overlapping PCR. The mutant was named as CCR7 MUT1/2. The 293T cells were co-transfected with miR-532-3p mimics/NC mimics and CCR7 WT (1 and 2)/CCR7 MUT (1 and 2). The 293T cells were purchased from Zhong Qiao Xin Zhou Biotechnology (Shanghai, China) and cultured in DMEM plus 10% FBS at 37°C containing 5% CO_2_ cell incubator. The binding activity of miR-532-3p was quantitatively assessed by calculating the normalized luciferase activity (fly luciferase activity/renilla luciferase activity).

### Immunofluorescence Assay

The CAL-27 cells were incubated with CCL21 (350 ng/ml) and the immunofluorescence assay was performed at the 1st or 60th minute, respectively. In brief, 4% paraformaldehyde was used to fix the cells. Following, the cells were permeabilized with 0.1% TritonX-100. Then cells were incubated with the primary antibody CCR7 antibody after blocking with serum. The photos were obtained *via* a fluorescent microscope at 600×.

### Xenograft Experiment

For the *in vivo* tumor growth assay, the nude mice were ordered from the Beijing HFK Bioscience. All animal experiments were approved by the Ethics Review Committee of School of Stomatology, China Medical University. The nude mice were randomly divided into two groups, Control and Pre-miR-532. The CAL-27 cells transfected with pre-miR-532 were incubated in a cell incubator at 37°C in 5% CO_2_. Complete medium with G418 (Sigma, USA) was used to select clones of CAL-27. The monoclonal culture was selected, the cultivation was further expanded, and the stable cell lines were established. The CAL-27 cells overexpressing pre-miR-532 (5×10^6^) were subcutaneously injected into each nude mouse in the Pre-miR-532 group. Tumor volume was measured once every 3 days. The nude mice were intraperitoneally injected 200 mg/kg sodium pentobarbital after 28 d. The tumors were excised and frozen in liquid nitrogen, and then stored at −70°C for further analysis.

### Pathological Analysis

HE staining. Tumor tissue samples removed from nude mice were fixed in 4% paraformaldehyde and embedded in paraffin. Paraffin sections were cut to a thickness of 5 μm and stained with hematoxylin and eosin for histological evaluation.

Immunohistochemistry. Paraffin sections were incubated with 3% H_2_O_2_ for 15 min, followed blocking goat serum (Solarbio). Then, they were incubated overnight at 4°C with the Ki-67 antibody (Abcam, UK). On the second day, they were further probed with the corresponding horseradish-peroxidase-conjugated goat anti-rabbit antibody (ThermoFisher, USA) at 37°C for 1 h. After DAB color development and hematoxylin counterstaining, the sections was observed.

Tunel assay. Paraffin sections were permeabilized using 0.1% Triton X-100 (Beyotime), blocked with 3% H_2_O_2_, and marked with converter-POD accordong to the manufacturer’s instructions (In Situ Cell Death Detection Kit, Roche, Germany).

### Statistical Analysis

Statistical analysis was conducted using the GraphPad Prism 7.0. Results were presented as mean ± SD of at least three different experiments performed in triplicate. Student’s t-tests or one-way ANOVA plus multiple comparisons combined with Bonferroni’s *post-hoc* test were utilized to evaluate the difference between groups. A value of P < 0.05 was considered statistically significant.

## Results

### MiR-532-3p Is Down-Regulated and CCR7 is Up-Regulated in TC Tissues

**Figure 1:**
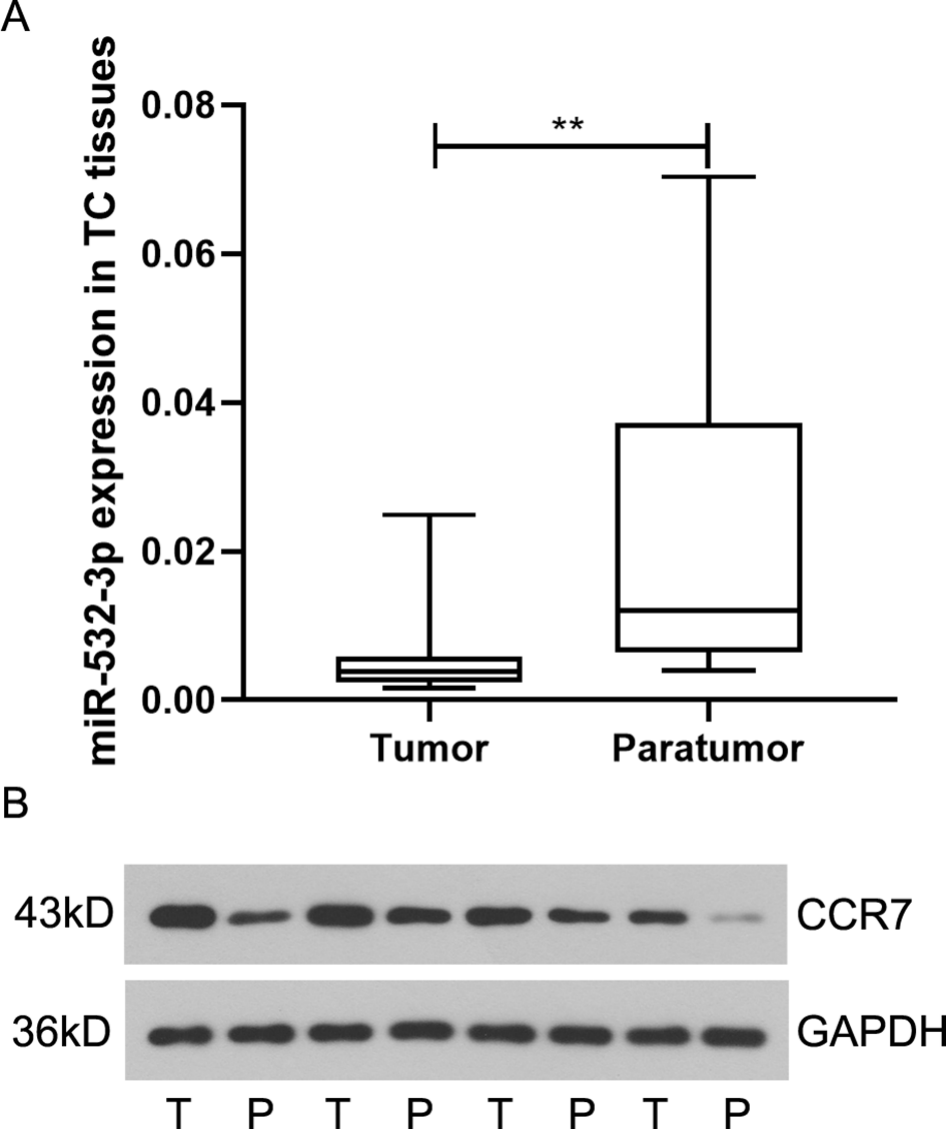
MiR-532-3p is down-regulated in tongue cancer (TC). **(A)** Expression of miR-532-3p in paired TC tissues and paratumor tissues (n = 23). **(B)** Western blot analysis of CCR7 in paired TC tissues and paratumor tissues. ** means p < 0.01.

To characterize miR-532-3p and CCR7 expression in TC, tissues containing 23 paired tumor and paratumor samples of TC were used for PCR and western blot analysis. From the results of real-time PCR, miR-532-3p transcript levels were found to decrease in tumor samples compared with those in paratumor samples (**Figure 1A**). On the contrary, CCR7 showed a higher expression in tumor samples at the protein level (**Figure 1B**). Please see detailed result in **Supplementary Materials (S1)**.

### Overexpression of miR-532-3p Inhibits Cell Proliferation and Facilitates Apoptosis in TSCC

**Figure 2:**
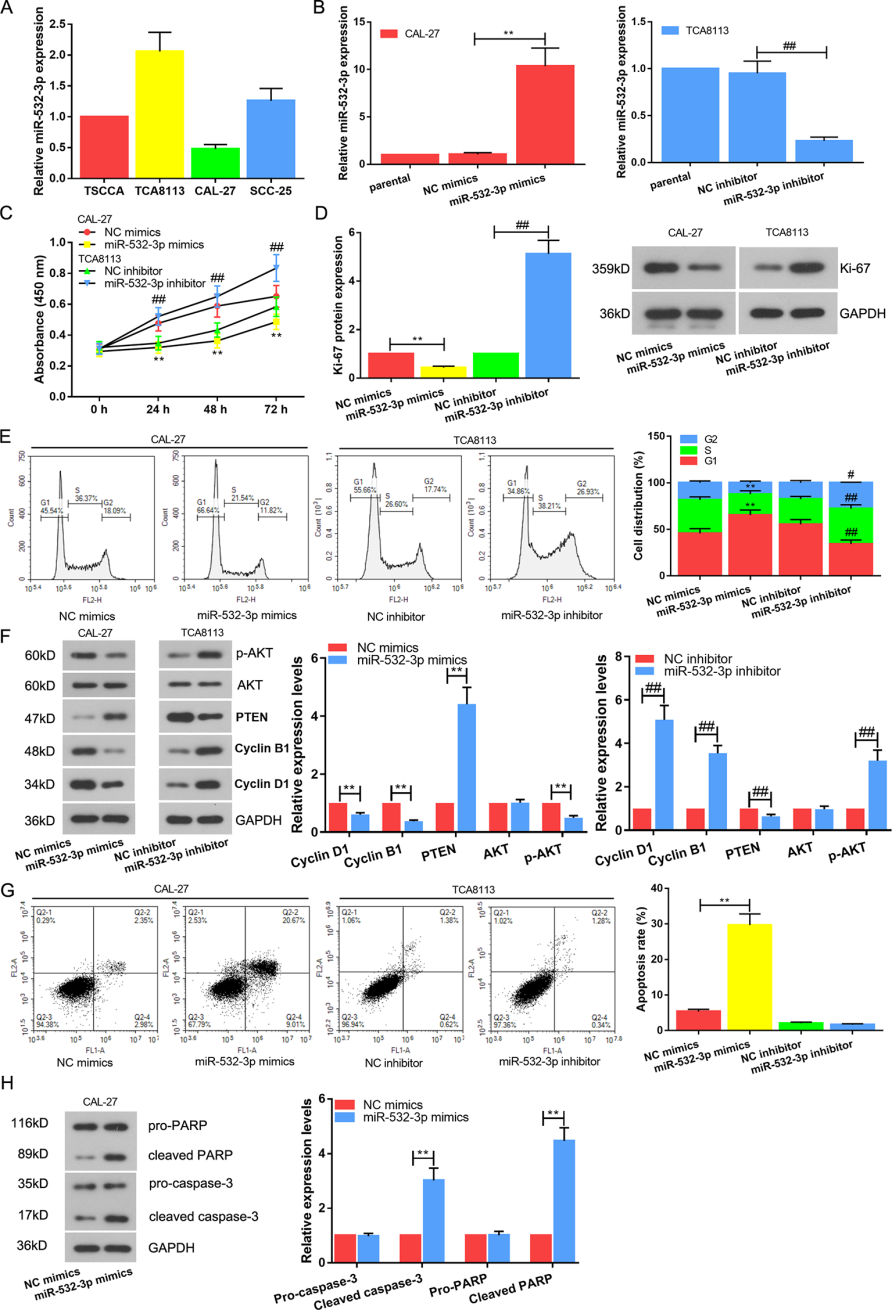
Overexpression of miR-532-3p inhibits cell proliferation and facilitates apoptosis in TSCC. **(A)** Expression of miR-532-3p was detected *via* real-time PCR in four types of TC cells, TSCCA, TCA8113, CAL-27, and SCC-25. Relatively highest and lowest miR-532-3p expression were in TCA8113 and CAL-27 cells, respectively. **(B)** Expression of miR-532-3p was increased after transfection miR-532-3p mimics in CAL-27 cell. When TCA8113 was transfected with miR-532-3p inhibitor, the expression of miR-532-3p was decreased. Cell proliferation was measured using CCk-8 assay **(C)**, Ki-67 protein expression analysis **(D)** and cell cycle detection **(E, F)**. **(G)** Promotion of apoptosis by overexpression of miR-532-3p. TCA8113 and CAL-27 cells were transfected for 48 h and apoptotic cells were monitored with flow cytometry after Annexin V and PI staining **(H)**. Apoptosis-related proteins (caspase-3 and PARP) were tested by western blot. **p < 0.01 vs. miR-532-3p mimics; #p < 0.05, ##p < 0.01 vs. miR-532-3p inhibitor.

The observation that the overexpression of miR-532-3p was correlated with tumor further inspired us to examine the functional role of miR-532-3p in TSCC cell lines. Expression of miR-532-3p was detected *via* real-time PCR in four types of TC cells, TSCCA, TCA8113, CAL-27, and SCC-25. As shown in **Figure 2A**, miR-532-3p expression level was lowest and highest in the CAL-27 and TCA8113, respectively. Next, miR-532-3p mimics and miR-532-3p inhibitor were transfected into CAL-27 and TCA8113, respectively. Transfection efficiency was analyzed. Expression of miR-532-3p was increased after transfection miR-532-3p mimics in CAL-27 cell. When TCA8113 was transfected with miR-532-3p inhibitor, the expression of miR-532-3p was decreased (**Figure 2B**). CCK-8 assays indicated that overexpression of miR-532-3p suppressed TSCC cell proliferation, whilst down regulation of miR-532-3p facilitated TSCC cell proliferation (**Figure 2C**, P < 0.01). The similar results were exhibited in Ki-67 expression (**Figure 2D**). Furthermore, overexpression of miR-532-3p significantly enhanced the percentage of cells in the G1 phase, while it reduced the percentage of cells in the G2 phase, which was determined by flow cytometry (**Figure 2E**). The protein levels of cyclin D1, cyclin B1 and p-AKT were decreased, simultaneously PTEN expression was increased after miR-532-3p overexpression (**Figure 2F**). In contrast, knocking down miR-532-3p promoted cyclin D1, cyclin B1 and p-AKT levels, whilst suppressed PTEN level and TSCC the percentage of cells in the G1 phase. Furthermore, apoptotic cells also were monitored with flow cytometry after Annexin V and PI staining (**Figure 2G**). Overexpression of miR-532-3p accelerated cell apoptosis. In addition, apoptosis-related proteins, caspase-3 and PARP, were increased (**Figure 2H**).

### Overexpression of miR-532-3p Suppresses Migration and Invasion of TSCC cells *In Vitro*

**Figure 3:**
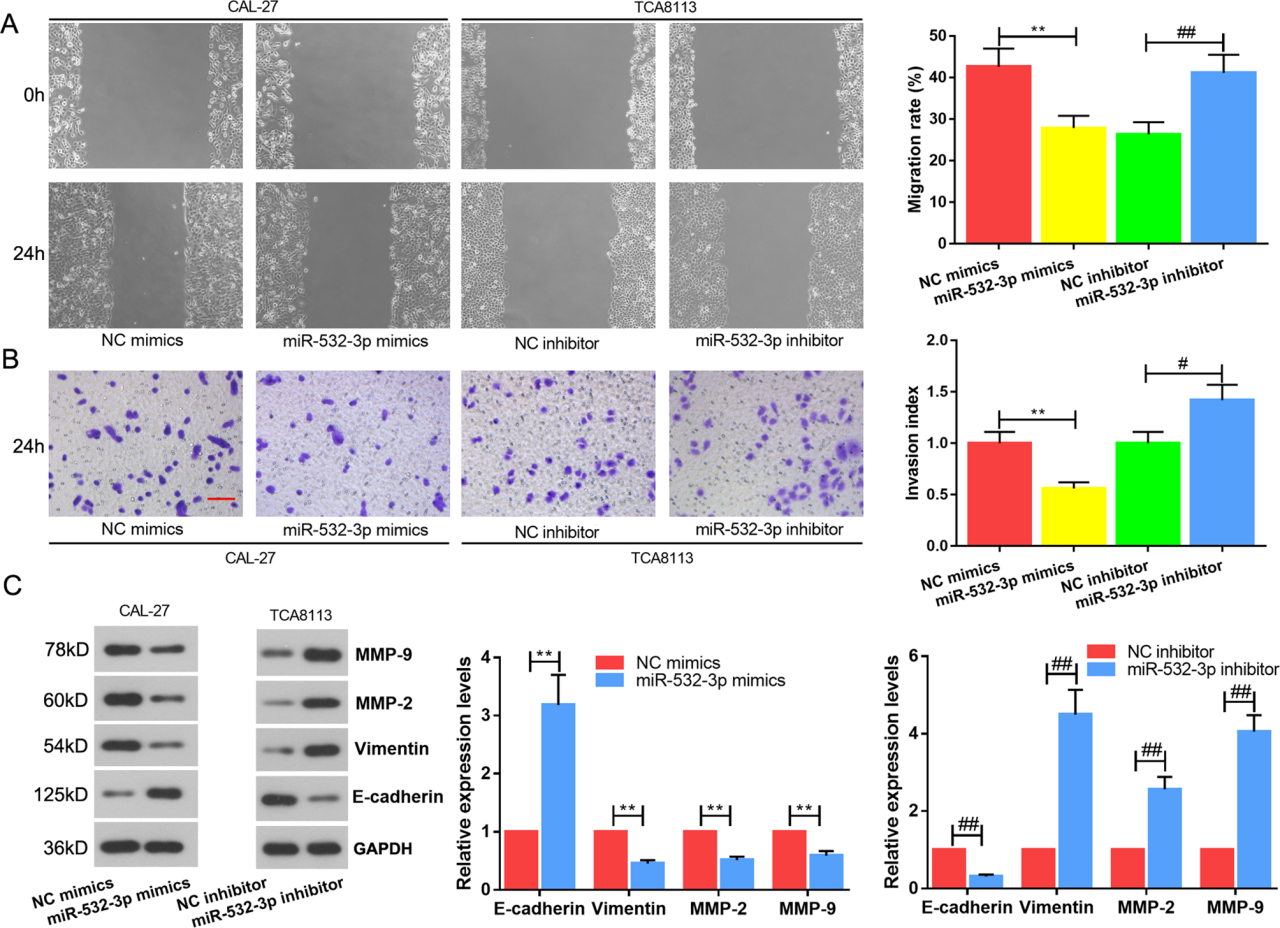
Overexpression of miR-532-3p suppresses migration and invasion of TSCC cells *in vitro*. Wound healing **(A)** and transwell **(B)** assays of TCA8113 and CAL-27 cells after transfection. **(C)** EMT protein analysis. E-cadherin, vimentin, MMP-2 and MMP-9 were detected by western blot. Scale bar = 100 µM. **p < 0.01 vs. miR-532-3p mimics; #p < 0.05, ##p < 0.01 vs. miR-532-3p inhibitor.

Based on the above results, the migration, invasion and epithelial-to-mesenchymal transition (EMT) proteins of TSCC cells were performed. Wound healing (**Figure 3A**) and transwell (**Figure 3B**) assays revealed that overexpression of miR-532-3p repressed the cell migration and invasion abilities of TSCC cells, whereas miR-532-3p knockdown the accelerated these abilities. In the meantime, EMT-related proteins were detected *via* western blot (**Figure 3C**). MiR-532-3p mimics inhibited EMT—E-cadherin expression was upregulated, whilst vimentin, MMP-2 and MMP-9 were downregulated. However, miR-532-3p inhibitor reversed this variation. Altogether, the data demonstrate that miR-532-3p functions as a tumor suppressor in TSCC cell lines.

### CCR7 Is a Direct Target of miR-532-3p in TSCC

**Figure 4:**
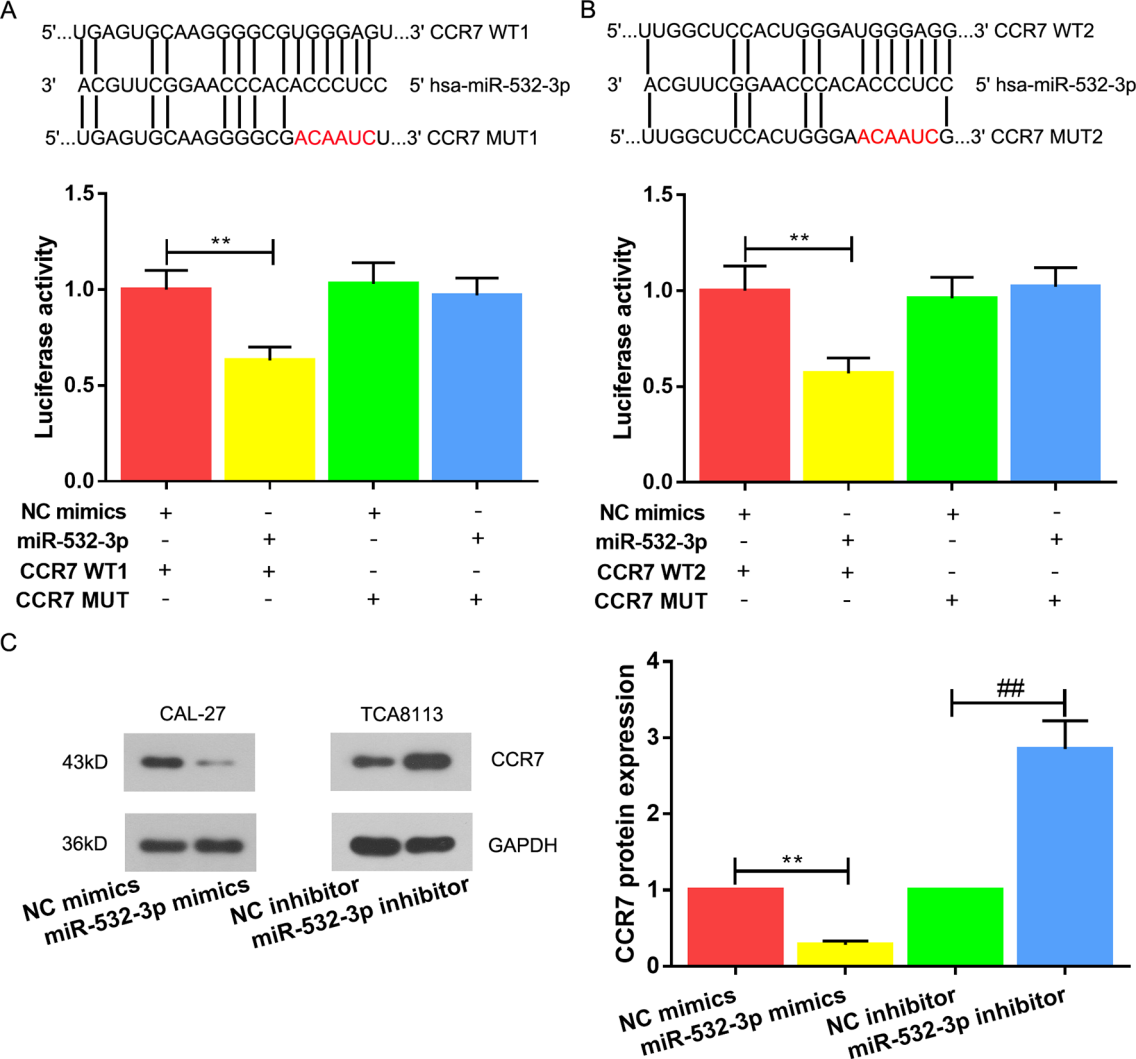
CCR7 is a direct target of miR-532-3p in TSCC. Putative wild-type (WT) and mutant (MUT) miR-532-3p binding sites in the 3’-UTR of CCR7. **(A,** **B)** Relative luciferase activities were analyzed in 293T cells co-transfected with WT (1 and 2) or MUT (1 and 2) reporter plasmids and miR-532-3p mimics or NC mimics. **(C)** CCR7 protein expression was ameliorated post-transfection miR-532-3p mimics in CAL-27 cell, whilst the expression of CCR7 was enhanced post-transfection miR-532-3p inhibitor. ** and ## means p < 0.01.

To further explore the downstream regulatory pathway of miR-532-3p in TSCC, we utilized publicly available bioinformatic algorithms to predict its downstream gene. The results predicted CCR7 as a potential regulator targeting miR-532-3p in TSCC. Luciferase reporter assays were carried out to corroborate this finding, a significant decrease in luciferase activities of the WT1 or WT2 were observed, whereas mutation in the putative binding site in the 3’UTR region of CCR7 abrogated the suppressive ability of miR-532-3p, verifying that miR-532-3p regulates CCR7 by directly binding to its 3’UTR region (**Figures 4A, B**, P < 0.01). Following, the decline of CCR7 in the miR-532-3p mimics group and the rise of CCR7 in the miR-532-3p inhibitor group confirmed this prediction (**Figure 4C**, P < 0.01).

### Enhanced Cell Proliferation, Migration And Invasion Induced by CCR7 Are Partly Restrained by miR-532-3p in TSCC Cell

**Figure 5:**
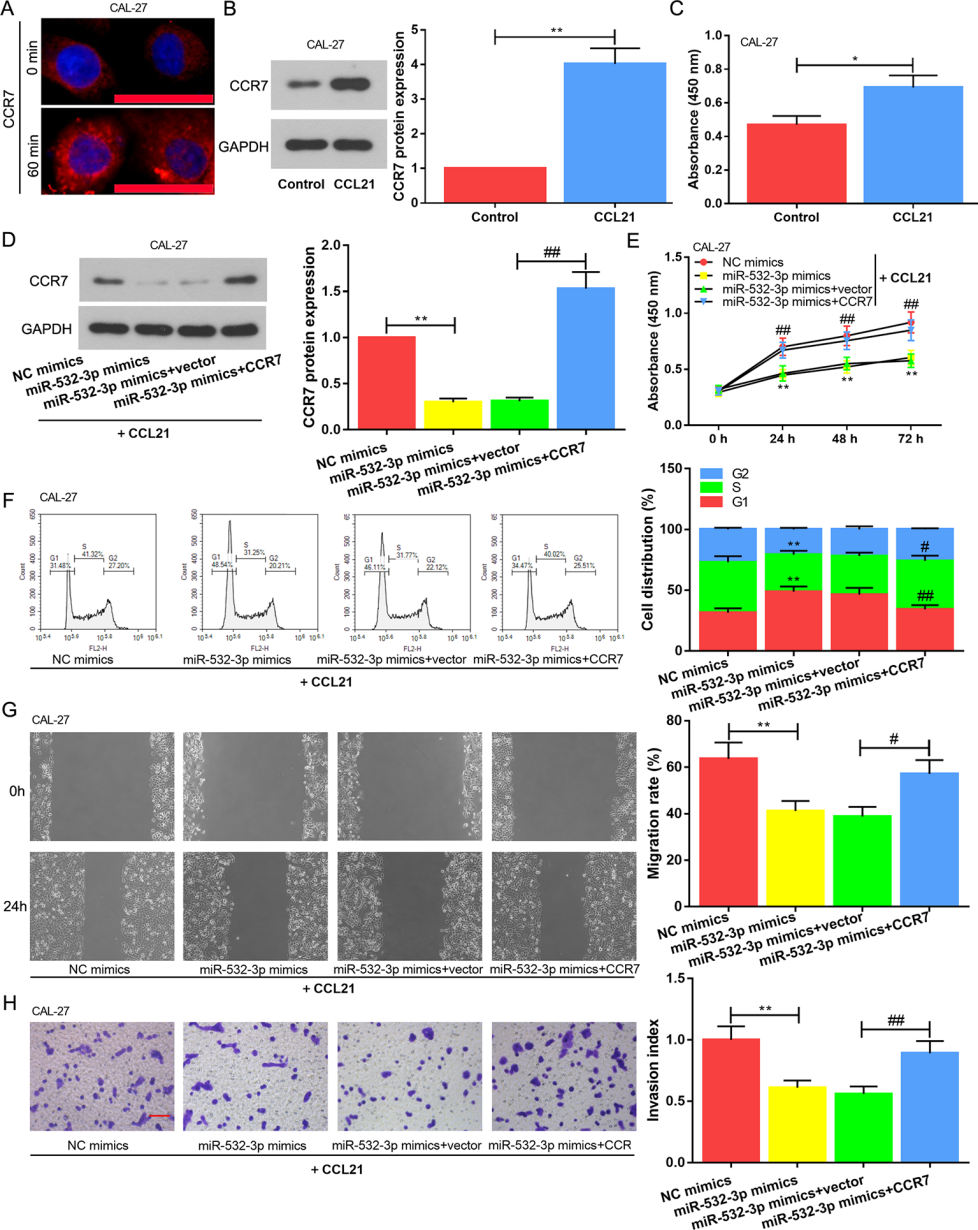
Enhanced cell proliferation, migration and invasion induced by CCR7 is partly restrained by miR-532-3p in TSCC cell. **(A)** The CAL-27 cells were incubated with 350 ng/ml CCL21 and the immunofluorescence assay was performed at the 1st or 60th minute, respectively. Scale bar = 50 µM. When CAL-27 cell was added into 350 ng/ml CCL21 for 48 h, western blot **(B)** and CCK-8 assay **(C)** were used for evaluating CCR7 level and cell proliferation, respectively. **(D)** Overexpressed miR-532-3p and CCR7 were co-transfected into CAL-27 cell, the expression level of CCR7 was detected. **(E)** MiR-532-3p mimics alleviated cell proliferation, however, CCR7 reversed this phenomenon. **(F)** Cell cycle arrest was disrupted due to the overexpression of CCR7. Cell migration **(G)** and invasion **(H)** were assessed *via* wound healing and transwell assays, respectively. Scale bar = 100 µM. * and # means p < 0.05; ** and ## means p < 0.01.

The CCL21 was added into the culture medium to ensure CCR7 activation. We estimated increased activity of the receptor *via* immunofluorescence assay. As shown in **Figure 5A**, CCR7 was localized on the cell surface of CAL-27 before ligand treatment (0 min), and the endocytosis was observed in response to CCL21 stimulation at the 60th min. This finding guaranteed that CCR7 was activated. Following, western blot assay revealed that CCR7 expression was increased in the CCL21 group than that in the Control group (**Figure 5B**). Meanwhile, OD values of CCL21 group was obviously elevated (**Figure 5C**). Thus, the cells which were co-transfected with miR-532-3p mimics and CCR7 were cultured in the present of CCL21. CCR7 level was initial evaluated. It was found that the CCR7 expression (**Figure 5D**) and OD values (**Figure 5E**) in the miR-532-3p mimics+vector were observably alleviated compared with those in the miR-532-3p mimics+CCR7. Moreover, the cell cycle assay indicated that the CCR7 reversed the role of miR-532-3p mimics in blocking cell cycle in G1 phase (**Figure 5F**). As the same time, migration and invasion induced by CCR7 were partly restrained by miR-532-3p overexpression (**Figures 5G, H**, P < 0.05).

### MiR-532-3p Attenuates Tumourigenesis *In Vivo*

**Figure 6:**
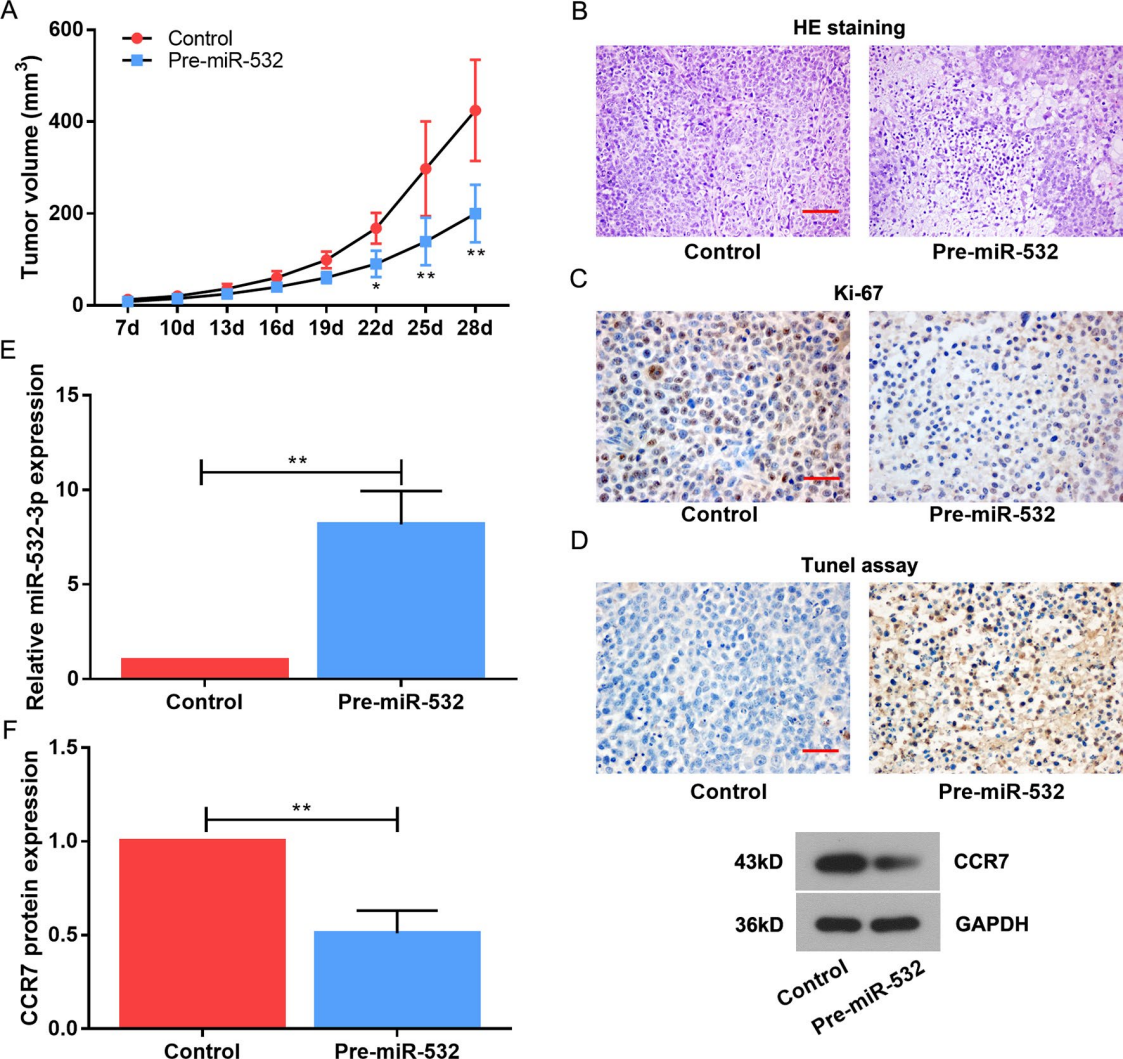
MiR-532-3p hinders TSCC cell proliferation and simulates TSCC cell apoptosis *in vivo*. The tumor xenografts of CAL-27 in subcutaneously injected nude mice. **(A)** Data points are presented as mean tumor volume (mm^3^). **(B)** HE staining was used to observe the tumor physiopathologic variation. Scale bar = 100 µM. **(C)** The immunohistochemistry assay on Ki-67. Scale bar = 50 µM. **(D)** The tunel assay on cell apoptosis. Scale bar = 50 µM. **(E)** MiR-532-3p expression was measured using real-time PCR. **(F)** The CCR7 level was reduced in pre-miR-532-overexpressed nude mice. * means p < 0.05; ** means p < 0.01.

To further validate the growth-suppressive function of miR-532-3p, we performed a tumourigenesis experiment in a xenograft tumor model. In total, the CAL-27 cell line transfected by pre-miR-532-3p-overexpressing plasmid were transplanted subcutaneously into nude mice. As expected, tumor volumes were saliently reduced derived from pre-miR-532-3p-overexpressed CAL-27 cells versus those from the Control group (**Figure 6A**). The HE staining showed that the tumor cells of the tumor-forming tissue in Control were dense and in good shape, the nuclear staining was clear, and the cytoplasm was uniformly stained. A large number of apoptotic and necrotic areas were observed in the pre-miR-532 group, and cytoplasm was lightly stained in those areas (**Figure 6B**). In addition, the positive rate of Ki-67 label was higher than that of pre-miR-532 group from the immunohistochenistry assay (**Figure 6C**). Tunel assay showed that a mass of apoptotic cells in the tissue of pre-miR-532 group, however, few apoptotic cells in the Control group (**Figure 6D**). The upregulated miR-532-3p expression and downregulated CCR7 were confirmed by real-time PCR and western blot in the pre-miR-532-overexpressed nude mice tissues (**Figures 6E, F**).

## Discussion

TSCC is one of the most common and malignant types of oral cancer because the tongue is an active organ with an abundant blood supply (Schwam and Judson, 2016). Patients with systematic therapy have a poor prognosis. Therefore, targeted therapy opens a window for preventing the occurrence and development of TSCC. A good deal of evidence indicates that miR-532 participates in many human cancers. Nevertheless, the biological roles and molecular mechanisms of miR-532-3p, whether it facilitates or abrogates tumor progression in TSCC, have not been clarified. In this study, we first identified that miR-532-3p expression was ameliorated in TC tissues compared to paratumor tissues. In addition, miR-532-3p not only significantly inhibited cell proliferation, migration, invasion, and promoted apoptosis *in vitro* by targeting the 3’-UTR of CCR7, but it also suppressed tumourigenicity *in vivo*.

As described before, miR-532 is differentially expressed in several types of cancers. For instance, miR-532 has been regarded as a tumor suppressor and was studied in ovarian cancer, bladder cancer and colorectal cancer (CRC) (Wang et al., 2016; Song et al., 2018; Xie et al., 2019). In a study based on 58 paired CRC tissues, it is reported that miR-532 is downregulated and an independent indicator among patients. It can potently inhibit CRC cell proliferation and metastasis, increase cell apoptosis *in vitro* and reduce tumor growth *in vivo* by directly targeting IGF-1R and inactivating the PI3K/Akt signaling pathway (Song et al., 2018). These results support our data. Our data demonstrated that the overexpression of miR-532 enhanced expression of PTEN and suppressed AKT phosphorylation. The PTEN gene, a tumor suppressor, is necessary for AKT activation (Dahia, 2000). AKT signaling pathway activates many downstream cellular processes, including cell proliferation, migration, invasion and apoptosis (Gan and Zhang, 2009). When the PTEN is activated, the cell cycle is blocked in the G1 phase and the tumor formation and progression are inhibited (Okumura et al., 2006). Similarly, miR-29b overexpression suppresses the proliferation and cell-cycle progression of TSCC cells, which may be due to the fact that miR-29b could inhibit AKT phosphorylation in a PTEN-mediated manner (Jia et al., 2014). In addition, aberrantly highly expressed miR-532 is also observed in triple-negative breast cancer (Chang et al., 2015) and gastric cancer (Xu et al., 2016). It is reported that miR-532-5p functions as an oncogenic miRNA in gastric cancer cells by targeting RUNX3 at both transcriptional and translational level *in vitro* and *in vivo*. These findings suggest that miR-532 expression in various cancers may be tissue-specific, so miR-532 may be a new and effective method for diagnosing and treating patients with specific tumor types.

Cancer metastasis demands cancer cell invasion into the stroma, cancer cell migration and invasion into the vessels, as well as cancer cell proliferation in the lymph nodes (Hart et al., 1989). The beginning of the invasion and metastasis is caused by EMT (Kalluri and Weinberg, 2009). EMT refers to the loss of carcinoma epithelial phenotype and the acquisition of mesenchymal-associated features (Zeisberg and Neilson, 2009). TC is often followed with lymph node metastasis, and drug-resistant cancer cells in TSCC patients undergo EMT. Therefore, controlling the EMT for cancer treatment, including TSCC, is imperative. Our data reveal that the upregulation of miR-532-3p could significantly increase the level of the epithelial marker E-cadherin and decrease the mesenchymal marker vimentin as well as attenuate the expression of MMP-2 and MMP-9. These imply that miR-532-3p might reverse the EMT process to suppress cell invasion and metastasis. Meanwhile, Zhang et al. also reports that urothelial cancer associated 1 knockdown significantly suppresses TGFβ1-induced TC cell invasion and EMT by decreasing vimentin and increasing E-cadherin (Zhang et al., 2019). Shi et al. discovers that the EMT of TSCC cells is inhibited by ameliorating the expressions of MMP-2 and MMP-9 (Shi et al., 2018). All these data support our point.

Moreover, the chemokine receptor CCR7 is necessary for the regulation of migratory speed and plays an important role in cancer metastasis (Riol-Blanco et al., 2005; Legler et al., 2014). For example, CCR7 has been demonstrated as a biomarker that can predict lymph node metastases in breast cancer, and as a metastasis and prognosis indicator in patients with esophageal carcinoma (Liu et al., 2013). Related study identifies positive correlations between the CCR7 expression and lymphatic endothelial markers in the analyzed panel of breast cancer tissues (Tutunea-Fatan et al., 2015). The overexpression of CCR7 promotes the capacity of EMT cells to migrate toward CCL21-expressing tissue and to disseminate through the lymphatic system (Pang et al., 2016). *In vivo* studies show the knockdown of CCL21 in secondary lymphoid organs leads to the decrease of metastatic tumor formation, since this reduces both the chemotactic and antiapoptotic effects of CCR7-expressing tumor cells (Wang et al., 2008). CCL21 is constitutively expressed by the lymphatic endothelium of multiple organs, high endothelial venules of lymph nodes and Peyer’s patches, as well as stromal cells in T cell rich areas of lymph nodes, and spleen (Tutunea-Fatan et al., 2015). The extensive physiological distribution of the CCL21/CCR7 axis and its complex and multifaceted role in lymph node transport may be one of the reasons why the CCL21/CCR7 axis is considered to be a viable candidate axis for TSCC treatment. Therefore, based on bioinformatics analysis, this study further verifies that miR-532-3p restrains malignant behaviors of TSCC cell *via* targeting CCR7. See the mechanism figure in **Supplementary Materials (S2)**.

## Conclusion

Collectively, these findings add newer insights into the multifaceted role played by the miR-532-3p, prompting for the first time towards the involvement of miR-532-3p in the complex mechanics of TSCC-induced malignant behaviors. In addition, it is suggested that miR-532-3p inhibits cell proliferation, migration and invasion *via* targeting CCR7. Therefore, the miR-532-3p/CCR7 axis might be the new biomarkers and therapeutic targets for TSCC treatment.

## Data Availability

The raw data supporting the conclusions of this manuscript will be made available by the authors, without undue reservation, to any qualified researcher.

## Ethics Statement

For human subjects: This study was carried out in accordance with the recommendations of School of Stomatology, China Medical University with written informed consent from all subjects. All subjects gave written informed consent in accordance with the Declaration of Helsinki. The protocol was approved by the School of Stomatology, China Medical University. For animal subjects: This study was carried out in accordance with the recommendations of School of Stomatology, China Medical University. The protocol was approved by the School of Stomatology, China Medical University.

## Author Contributions

The study was conceived by ZX. The manuscript was written by CF. CF, HS, SY, XS, QX, SW, WD, EZ and CS performed experiments and took part in the analysis of data. ZX polished the manuscript.

## Funding

This study was supported by grants from the Foundation of Education Bureau of Liaoning Province (No. L2014317) and the Natural Science Foundation of Liaoning Province (No. 2014021096).

## Conflict of Interest Statement

The authors declare that the research was conducted in the absence of any commercial or financial relationships that could be construed as a potential conflict of interest.

## References

[B1] Chang Y. Y. Kuo W. H. Hung J. H. Lee C. Y. Lee Y. H. Chang Y. C. (2015). Deregulated microRNAs in triple-negative breast cancer revealed by deep sequencing. Mol. Cancer 14, 36. 10.1186/s12943-015-0301-9 25888956 PMC4351690

[B2] Dahia P. L. (2000). PTEN, a unique tumor suppressor gene. Endocr. Relat. Cancer 7, 115–129. 10.1677/erc.0.0070115 10903528

[B3] Gan Y. H. Zhang S. (2009). PTEN/AKT pathway involved in histone deacetylases inhibitor induced cell growth inhibition and apoptosis of oral squamous cell carcinoma cells. Oral Oncol. 45, e150–e154. 10.1016/j.oraloncology.2009.05.563 19574087

[B4] Gorsky M. Epstein J. B. Oakley C. Le N. D. Hay J. Stevenson-Moore P. (2004). Carcinoma of the tongue: a case series analysis of clinical presentation, risk factors, staging, and outcome. Oral Surg. Oral Med. Oral Pathol. Oral Radiol. Endod. 98, 546–552. 10.1016/j.tripleo.2003.12.041 15529126

[B5] Han J. Wang F. Lan Y. Wang J. Nie C. Liang Y. (2019). KIFC1 regulated by miR-532-3p promotes epithelial-to-mesenchymal transition and metastasis of hepatocellular carcinoma *via* gankyrin/AKT signaling. Oncogene 38, 406–420. 10.1038/s41388-018-0440-8 30115976 PMC6336682

[B6] Hart I. R. Goode N. T. Wilson R. E. (1989). Molecular aspects of the metastatic cascade. Biochim. Biophys. Acta 989, 65–84. 10.1016/0304-419X(89)90035-8 2665818

[B7] Jia L. F. Huang Y. P. Zheng Y. F. LYU M. Y. Wei S. B. Meng Z. (2014). miR-29b suppresses proliferation, migration, and invasion of tongue squamous cell carcinoma through PTEN-AKT signaling pathway by targeting Sp1. Oral Oncol. 50, 1062–1071. 10.1016/j.oraloncology.2014.07.010 25127200

[B8] Kalluri R. Weinberg R. A. (2009). The basics of epithelial-mesenchymal transition. J. Clin. Invest. 119, 1420–1428. 10.1172/JCI39104 19487818 PMC2689101

[B9] Kokemueller H. Rana M. Rublack J. Eckardt A. Tavassol F. Schumann P. (2011). The hannover experience: surgical treatment of tongue cancer–a clinical retrospective evaluation over a 30 years period. Head Neck Oncol. 3, 27. 10.1186/1758-3284-3-27 21600000 PMC3123311

[B10] Legler D. F. Uetz-Von Allmen E. Hauser M. A. (2014). CCR7: roles in cancer cell dissemination, migration and metastasis formation. Int. J. Biochem. Cell. Biol. 54, 78–82. 10.1016/j.biocel.2014.07.002 25019368

[B11] Lin X. J. He C. L. Sun T. Duan X. J. Sun Y. Xiong S. J. (2017). hsa-miR-485-5p reverses epithelial to mesenchymal transition and promotes cisplatin-induced cell death by targeting PAK1 in oral tongue squamous cell carcinoma. Int. J. Mol. Med. 40, 83–89. 10.3892/ijmm.2017.2992 28535002 PMC5466395

[B12] Liu X. Y. Song L. Wang Z. (2013). CCR7: a metastasis and prognosis indicator of postoperative patients with esophageal carcinoma. Hepatogastroenterology 60, 747–750. 10.5754/hge12846 23165192

[B13] Maruyama T. Nishihara K. Umikawa M. Arasaki A. Nakasone T. Nimura F. (2018). MicroRNA-196a-5p is a potential prognostic marker of delayed lymph node metastasis in early-stage tongue squamous cell carcinoma. Oncol. Lett. 15, 2349–2363. 10.3892/ol.2017.7562 29434944 PMC5778269

[B14] Okumura K. Mendoza M. Bachoo R. M. DePinho R. A. Cavenee W. K. Furnari F. B. (2006). PCAF modulates PTEN activity. J. Biol. Chem. 281, 26562–26568. 10.1074/jbc.M605391200 16829519

[B15] Pang M. F. Georgoudaki A. M. Lambut L. Johansson J. Tabor V. Hagikura K. (2016). TGF-beta1-induced EMT promotes targeted migration of breast cancer cells through the lymphatic system by the activation of CCR7/CCL21-mediated chemotaxis. Oncogene 35, 748–760. 10.1038/onc.2015.133 25961925 PMC4753256

[B16] Riol-Blanco L. Sanchez-Sanchez N. Torres A. Tejedor A. Narumiya S. Corbi A. L. (2005). The chemokine receptor CCR7 activates in dendritic cells two signaling modules that independently regulate chemotaxis and migratory speed. J. Immunol. 174, 4070–4080. 10.4049/jimmunol.174.7.4070 15778365

[B17] Schwam Z. G. Judson B. L. (2016). Improved prognosis for patients with oral cavity squamous cell carcinoma: analysis of the national cancer database 1998-2006. Oral Oncol. 52, 45–51. 10.1016/j.oraloncology.2015.10.012 26553389

[B18] Shang A. Lu W. Y. Yang M. Zhou C. Zhang H. Cai Z. X. (2018). miR-9 induces cell arrest and apoptosis of oral squamous cell carcinoma *via* CDK 4/6 pathway. Artif. Cells Nanomed. Biotechnol. 46, 1754–1762. 10.1080/21691401.2017.1391825 29073835

[B19] Shi B. Yan W. Liu G. Guo Y. (2018). MicroRNA-488 inhibits tongue squamous carcinoma cell invasion and EMT by directly targeting ATF3. Cell. Mol. Biol. Lett. 23, 28. 10.1186/s11658-018-0094-0 29946339 PMC6006839

[B20] Song Y. Zhao Y. Ding X. Wang X. (2018). microRNA-532 suppresses the PI3K/Akt signaling pathway to inhibit colorectal cancer progression by directly targeting IGF-1R. Am. J. Cancer Res. 8, 435–449. 29636999 PMC5883094

[B21] Tutunea-Fatan E. Majumder M. Xin X. Lala P. K. (2015). The role of CCL21/CCR7 chemokine axis in breast cancer-induced lymphangiogenesis. Mol. Cancer 14, 35. 10.1186/s12943-015-0306-4 25744065 PMC4339430

[B22] Wang C. Huang H. Huang Z. Wang A. Chen X. Huang L. (2011). Tumor budding correlates with poor prognosis and epithelial-mesenchymal transition in tongue squamous cell carcinoma. J. Oral Pathol. Med. 40, 545–551. 10.1111/j.1600-0714.2011.01041.x 21481005 PMC3135705

[B23] Wang F. Chang J. T. Kao C. J. Huang R. S. (2016). High expression of miR-532-5p, a tumor suppressor, leads to better prognosis in ovarian cancer both in vivo and in vitro. Mol. Cancer Ther. 15, 1123–1131. 10.1158/1535-7163.MCT-15-0943 26873729 PMC4873383

[B24] Wang J. Seethala R. R. Zhang Q. Gooding W. Van Waes C. Hasegawa H. (2008). Autocrine and paracrine chemokine receptor 7 activation in head and neck cancer: implications for therapy. J. Natl. Cancer Inst. 100, 502–512. 10.1093/jnci/djn059 18364504

[B25] Xia X. Liu K. Zhang H. Shang Z. (2015). Correlation between CCR7 expression and lymph node metastatic potential of human tongue carcinoma. Oral Dis. 21, 123–131. 10.1111/odi.12228 24528991

[B26] Xie X. Pan J. Han X. Chen W. (2019). Downregulation of microRNA-532-5p promotes the proliferation and invasion of bladder cancer cells through promotion of HMGB3/Wnt/beta-catenin signaling. Chem. Biol. Interact. 300, 73–81. 10.1016/j.cbi.2019.01.015 30639441

[B27] Xu X. Zhang Y. Liu Z. Zhang X. Jia J. (2016). miRNA-532-5p functions as an oncogenic microRNA in human gastric cancer by directly targeting RUNX3. J. Cell. Mol. Med. 20, 95–103. 10.1111/jcmm.12706 26515139 PMC4717862

[B28] Yang T. L. Wang C. P. Ko J. Y. Lin C. F. Lou P. J. (2008). Association of tumor satellite distance with prognosis and contralateral neck recurrence of tongue squamous cell carcinoma. Head Neck 30, 631–638. 10.1002/hed.20758 18213729

[B29] Zeisberg M. Neilson E. G. (2009). Biomarkers for epithelial-mesenchymal transitions. J. Clin. Invest. 119, 1429–1437. 10.1172/JCI36183 19487819 PMC2689132

[B30] Zhai W. Ma J. Zhu R. Xu C. Zhang J. Chen Y. (2018). MiR-532-5p suppresses renal cancer cell proliferation by disrupting the ETS1-mediated positive feedback loop with the KRAS-NAP1L1/P-ERK axis. Br. J. Cancer 119, 591–604. 10.1038/s41416-018-0196-5 30082686 PMC6162242

[B31] Zhang T. H. Liang L. Z. Liu X. L. Wu J. N. Su K. Chen J. Y. (2019). LncRNA UCA1/miR-124 axis modulates TGFbeta1-induced epithelial-mesenchymal transition and invasion of tongue cancer cells through JAG1/Notch signaling. J. Cell. Biochem. 120, 10495–10504. 10.1002/jcb.28334 30635938

