# Supplementary material for: MicroRNA-532-3p Suppresses Malignant Behaviors of Tongue Squamous Cell Carcinoma via Regulating CCR7
Source: Front Pharmacol. 2019 Aug 29;10:940. doi: 10.3389/fphar.2019.00940 (PMC6727182; doi:10.3389/fphar.2019.00940)
Supplement: Supplementary file 1 [file Table_1.docx]

Supplementary materials

MicroRNA-532-3p suppresses malignant behaviors of tongue squamous cell carcinoma via regulating CCR7

Cuijuan Feng^1^, Hyon Il So^2^, Shoucheng Yin^3^, Xingzhou Su^3^, Qiang Xu^3^, Simin Wang^3^, Weiyi Duan^3^, Enjiao Zhang^3^, Changfu Sun^3^, Zhongfei Xu^3, *^

^1^Department of Orthodontics, School of Stomatology, China Medical University, Shenyang 110002, People’s Republic of China

^2^Department of Oral and Maxillofacial Surgery, Pyongyang Medical College, Kim IL Sung University, Pyongyang 44-16-1, Democratic People’s Republic of Korea

^3^Department of Oral and Maxillofacial Surgery, School of Stomatology, China Medical University, Shenyang 110002, People’s Republic of China

^*^Corresponding author: Dr. Zhongfei Xu, Department of Oral and Maxillofacial Surgery, School of Stomatology, China Medical University, 117 North Nanjing Street, Shenyang 110002, People’s Republic of China

E-mail: xzf7090@outlook.com


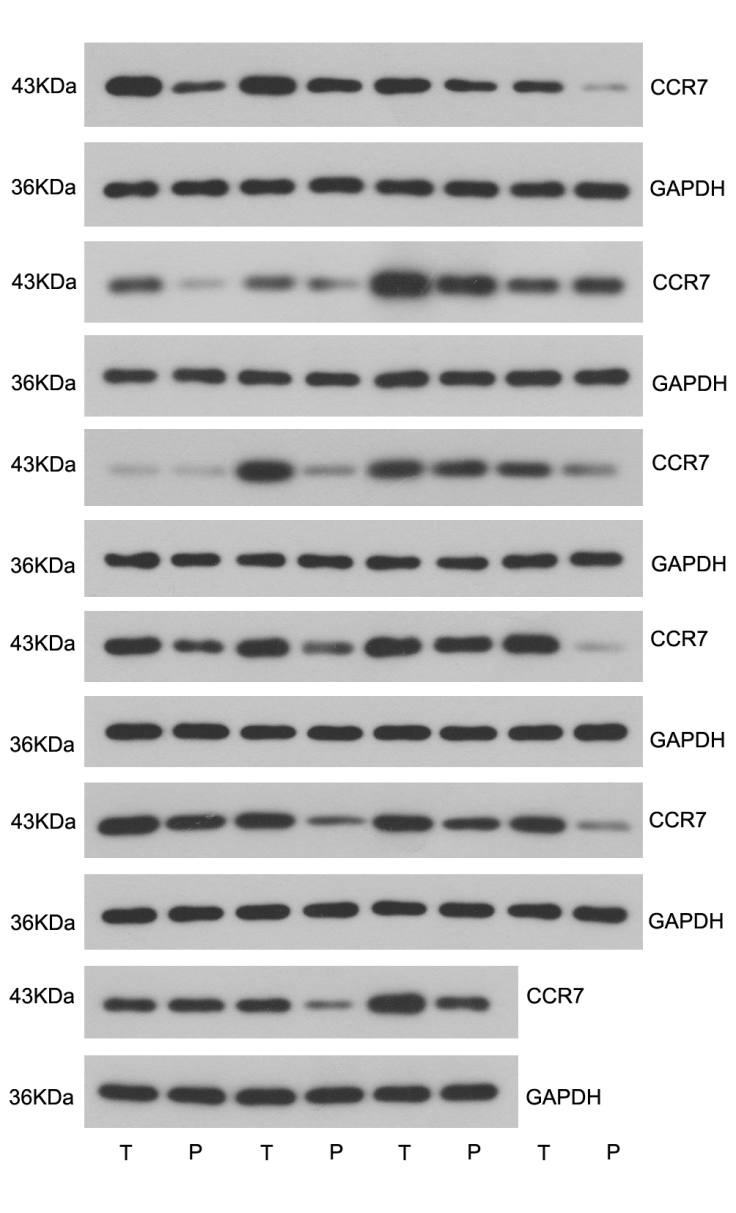


S1: Western blot analysis of CCR7 in paired TC tissues and paratumor tissues (n = 23).


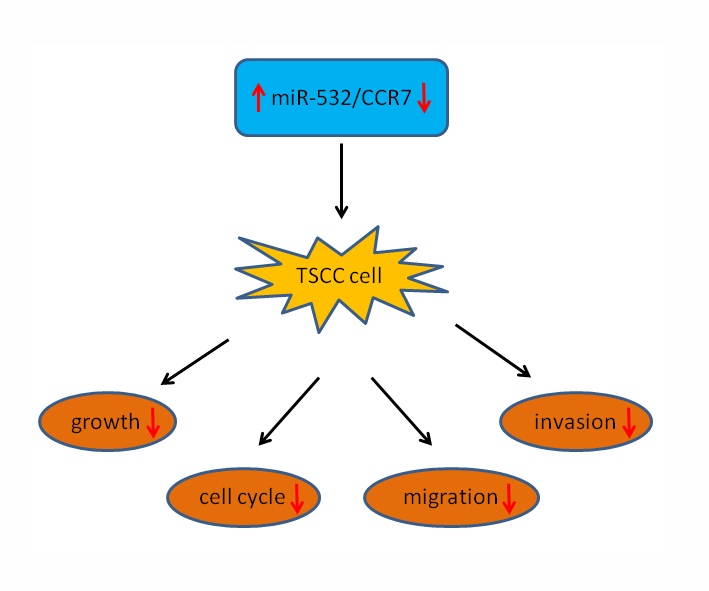


S2: The proposed mechanism of actions. MiR-532-3p targets CCR7, resulting in inhibition of CCR7. Following, it influences the biological features of TSCC cells, including proliferation, migration, and invasion.
